# Dietary supplementation of *Acer truncatum* leaf extract alleviates oxidative-stress induced impairment of ovarian function in laying hens via Nrf2-mediated antioxidant defense and VEGF-mediated angiogenesis

**DOI:** 10.1007/s44154-026-00329-x

**Published:** 2026-08-12

**Authors:** Kailong Qin, Junjie Ma, Minglu Gao, Yanli Liu, Xiaojun Yang

**Affiliations:** College of Animal Science and Technology, Northwest A&F University, Yangling, 712100 China

**Keywords:** Acer truncatum leaf extract, Oxidative stress, Angiogenesis, Ovary, Laying hens

## Abstract

This study aimed to evaluate the potential of Acer truncatum leaf extract (ATLE) to mitigate oxidative-stress–induced impairment of reproductive function in laying hens, with a focus on mechanisms involving antioxidant defense, angiogenesis, and ovarian microenvironment homeostasis. Oxidative stress was induced by tert-butyl hydroperoxide (tBHP) injection. Hens were randomly assigned to a control group (CON), an oxidative stress group (BCON), and an oxidative stress group supplemented with 0.6% ATLE (BATLE). Egg-laying performance, ovarian morphology, reproductive hormones, oxidative stress markers, and angiogenesis indicators were evaluated and integrated with transcriptomic analysis. The results were as follows: 1) Production and Morphology: ATLE supplementation alleviated (*P* < 0.05) the tBHP-induced decrease in egg laying rate and elevation in feed conversion ratio (FCR). It also mitigated (*P* < 0.05) reductions in the number of hierarchical follicles and the ovarian stroma index while restoring (*P* < 0.05) serum concentrations of LH and GH, thereby re-establishing reproductive endocrine balance. 2) Antioxidant Defense: ATLE activated (*P* < 0.05) Nrf2 and increased (*P* < 0.05) transcription of downstream antioxidant genes, including *SOD3*, *GPX3*, *PRDX4*, *GSR* and *CAT*, thereby enhancing (*P* < 0.05) the SOD and CAT activities and reducing (*P* < 0.05) MDA concentrations within both serum and ovarian tissues. 3) Vascular Network Support: ATLE attenuated (*P* < 0.05) the tBHP-induced downregulation of angiogenic factors, including protein levels of VEGF, ANGPT1, and HIF-1α and mRNA expression levels of *VEGFA*, *ANGPT1*, *ANGPT2*, *ITGA5*, and *MMP9*. 4) Microenvironment Remodeling: Transcriptomic integration revealed that ATLE-mediated repair overlapped (> 88%) with oxidative-stress–induced damage across functional modules. By reversing dysregulation of key genes such as *VEGFA*, *KDR*, and *FN1*, ATLE restored core pathways including focal adhesion and ECM–receptor interaction, and coordinated processes such as extracellular matrix, angiogenesis, and response to wounding, driving a systemic remodeling of the ovarian reproductive microenvironment toward homeostasis. In summary, this study demonstrated that ATLE activated multiple signaling axes, notably Nrf2 and VEGF. As a result, ATLE enhanced antioxidant defenses against oxidative damage and promoted vascular health and microenvironmental remodeling in the ovary. Together, these effects preserved the structural integrity and function of the laying hen reproductive system. These findings supported the potential of ATLE as a natural feed additive and provided a scientific rationale for using nutritional strategies to alleviate oxidative stress and improve reproductive health in laying hens.

**Graphical Abstract:**

**Figure:**
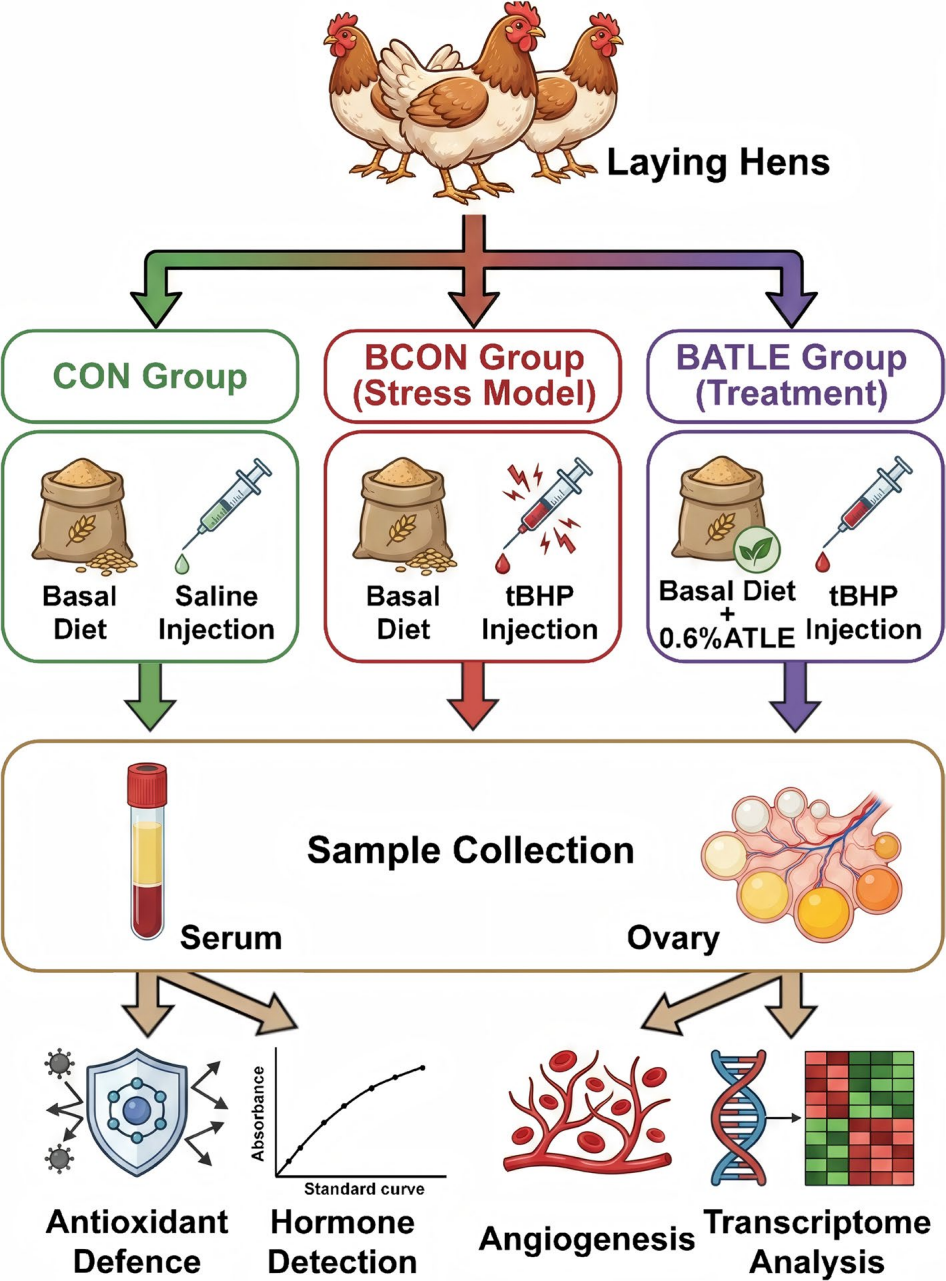

**Supplementary Information:**

The online version contains supplementary material available at 10.1007/s44154-026-00329-x.

## Introduction

The modern poultry industry faces a critical challenge in balancing high production efficiency with animal health and well-being (Humam et al. 2021; Gao et al. 2025; Xu et al. 2025). Nutritional strategies and functional feed additives exert broad systemic protective effects across multiple vital organs, including the liver and intestine, which are crucial for nutrient processing, metabolic homeostasis, barrier integrity, and egg synthesis (Huang et al. 2025; Shen et al. 2026; Yang et al. 2026). Building upon these systemic requirements, the avian ovary presents a uniquely dynamic physiological target. The avian ovary, which undergoes rapid follicle turnover and requires substantial energy for follicle recruitment and maturation, is especially vulnerable to oxidative imbalance. This imbalance accelerates reproductive aging and impairs the ovarian microvascular network (Zhao et al. 2023; Mu et al. 2025; Zahedi et al. 2025). Angiogenesis is essential for follicular development, nutrient delivery, and hormone transport (Xu et al. 2022a, b; Li et al. 2025a, b), and insufficient angiogenesis is a major contributor to follicular atresia (Li et al. 2020; Xu et al. 2022a, b; Li et al. 2025a, b). However, excessive reactive oxygen species (ROS) not only directly induce damage to vascular endothelial cells but also disrupt hypoxia signaling, which suppresses the expression of key angiogenic factors like VEGF and ANGPT1 (Wang et al. 2023; Qian et al. 2025). This oxidative damage causes severe microcirculatory disorders, depriving developing follicles of essential structural support and leading to follicular atresia and a subsequent decline in laying performance. Consequently, within the "One Health" framework, there is an urgent need to develop natural and sustainable feed additives that mitigate oxidative stress and promote ovarian vascular health (Jiang et al. 2025a, b).

Plant-derived polyphenols and flavonoids with multi-target biological activities are considered ideal candidates to address the above needs (Chen et al. 2022, 2023; Sookying et al. 2026). For example, catechins (Bertozzi-Matheus et al. 2024), curcumin (Tubsakul et al. 2021), astragaloside IV (Zhang et al. 2021) and chlorogenic acid (Ekowati et al. 2023; Bi et al. 2024) have been demonstrated to activate antioxidant pathways, modulate inflammatory responses, and protect vascular endothelial cells. *Acer truncatum* leaf extract (ATLE) represents a prominent example of such bioactive complexes, as it is exceptionally rich in a diverse array of polyphenols and flavonoids that synergistically combat oxidative damage (Fan et al. 2023; Liu et al. 2025a, b, c; Long et al. 2023; Liao and He 2025). Indeed, previous research has already confirmed the potent anti-inflammatory and antioxidant properties of ATLE in the intestinal tract of poultry (Liu et al. 2023). However, systematic evidence on whether ATLE could mitigate oxidative stress–induced declines in ovarian function by enhancing the critical processes of angiogenesis and/or tissue remodeling remains lacking.

It was hypothesized that ATLE would enhance systemic antioxidant defense in laying hens and protect ovarian function by promoting angiogenesis and preserving tissue integrity. An oxidative stress model was established using tert-butyl hydroperoxide (tBHP) in laying hens. The objectives of the study were to: (1) evaluate the impact of ATLE on egg-laying performance, ovarian tissue morphology, and serum reproductive hormone concentrations; (2) investigate ATLE's effects on ovarian antioxidant enzyme activities and the expression of related genes, particularly those within the Nrf2 pathway; (3) assess ATLE's influence on key factors of ovarian angiogenesis and their gene expression; and (4) integrate transcriptomic data to clarify the ATLE-mediated remodeling of the ovarian microenvironment, thereby identifying the core molecular regulatory networks and metabolic pathways involved.

## Results

### Production performance

**Fig. 1:**
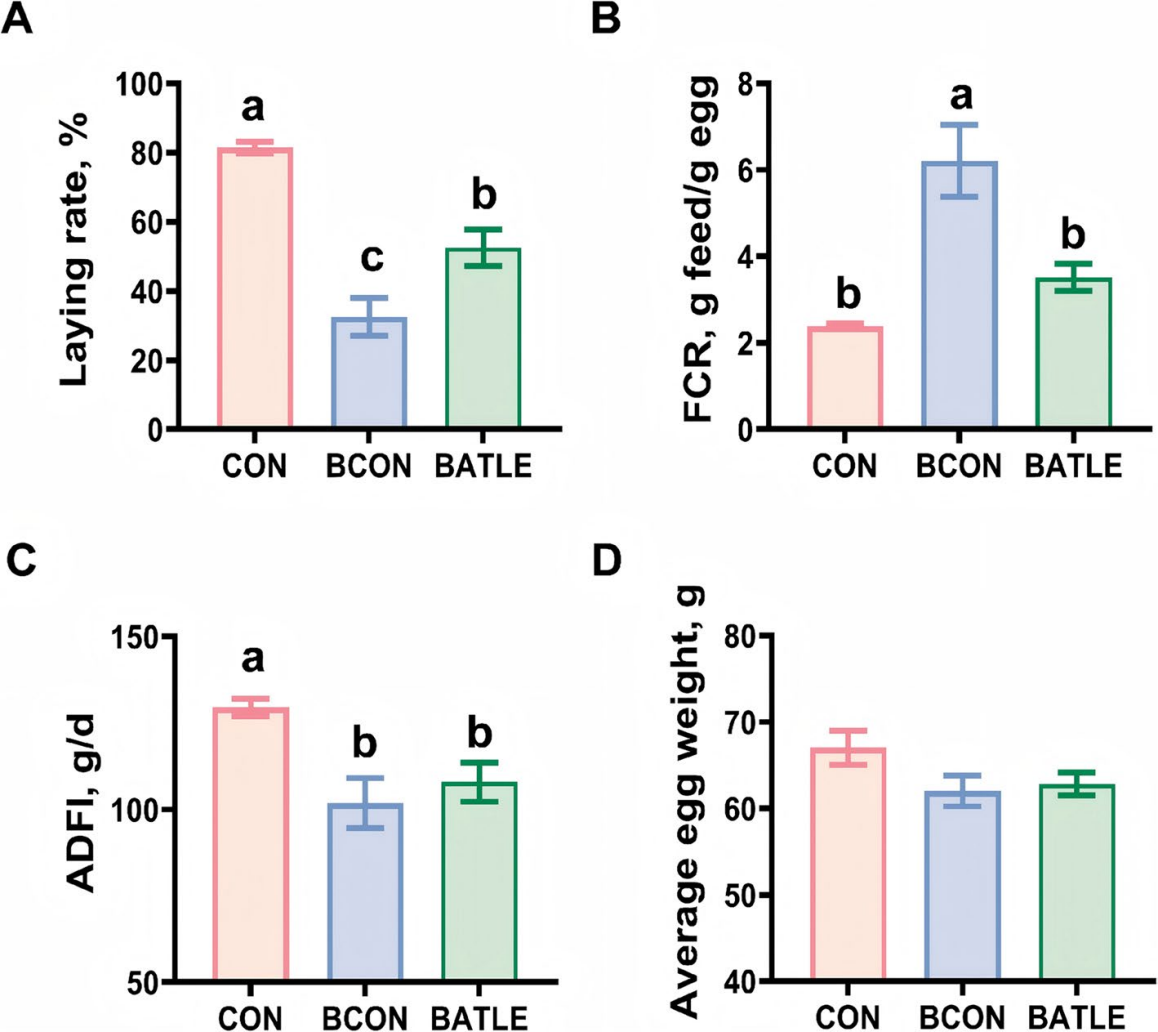
Effects of ATLE on laying performance in tBHP-challenged hens. **A** Laying rate, (**B**) FCR, (**C**) ADFI, (**D**) Average egg weight. Data are expressed as mean ± standard error (SEM). Different superscripts in indicate a significant difference (*P* < 0.05). Abbreviations: ATLE, Acer truncatum Bunge leaf extract; FCR, a ratio of the feed intake to the egg weight; ADFI, average daily feed intake

As shown in Fig. 1, compared with the unstressed CON group, the BCON group (injected with tBHP) exhibited a decrease (*P* < 0.05) in laying rate and ADFI (Fig. 1A, C), and an increase (*P* < 0.05) in FCR (Fig. 1B). However, ATLE supplementation alleviated (*P* < 0.05) the tBHP-induced decrease in egg laying rate and elevation in FCR. No differences (*P* > 0.05) were observed in average egg weight among treatments (Fig. 1D).

### Ovarian morphology

**Fig. 2:**
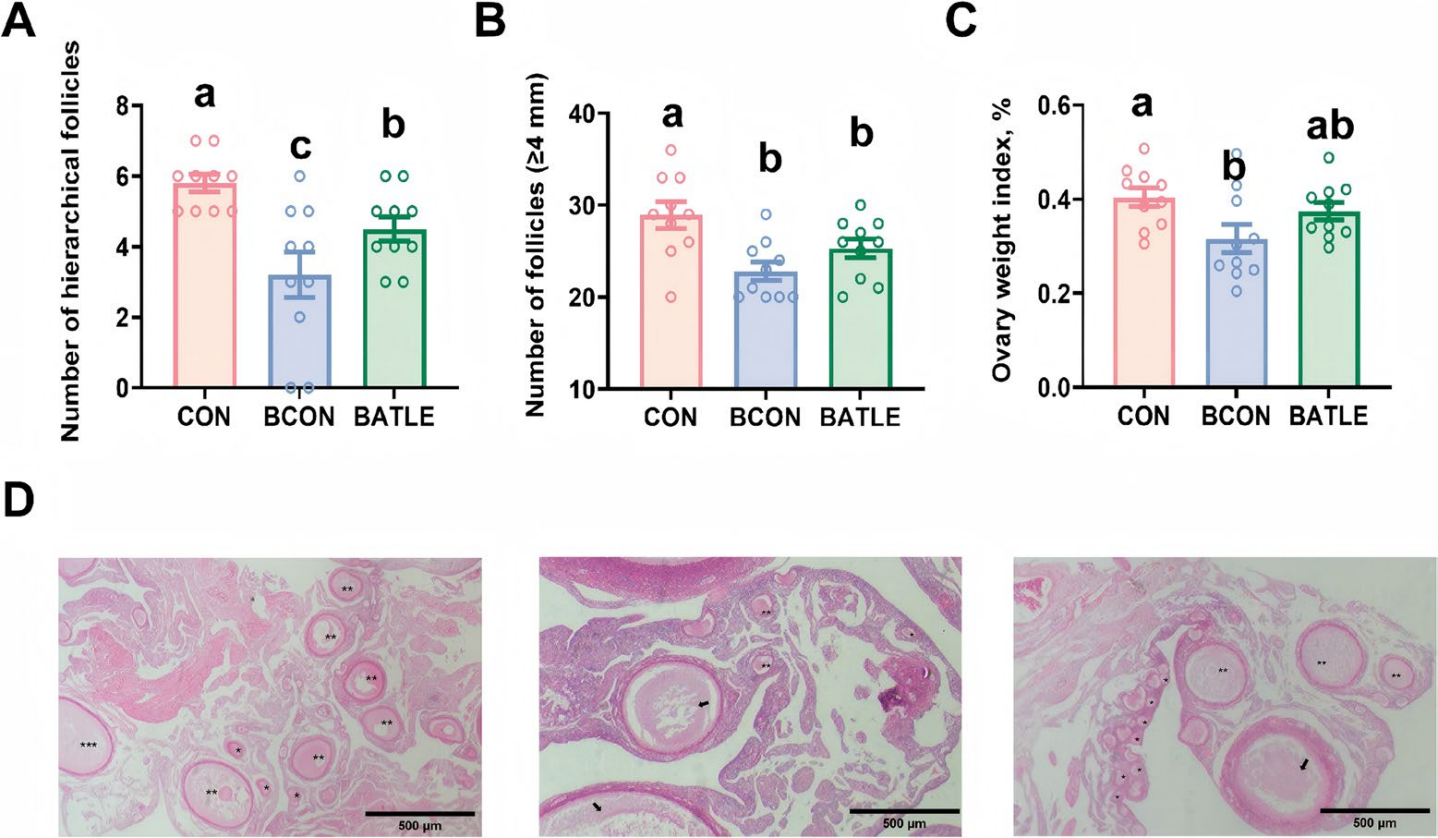
Effects of ATLE on follicular development and ovarian morphology in tBHP-challenged hens. **A** Number of hierarchical follicles, **B** Number of follicles (≥ 4 mm), (**C**) Ovary weight index, (**D**) H&E staining of ovarian tissue, (*) primary follicle, (**) secondary follicle, (***) mature follicle, and (→) atretic follicle. Data are expressed as mean ± standard error (SEM). Different superscripts indicate differences at *P* < 0.05. Abbreviations: ATLE, Acer truncatum Bunge leaf extract

Ovarian follicle development and morphology are shown in Fig. 2. The number of hierarchical follicles, small follicles (≥ 4 mm), and the ovarian stroma index were decreased (*P* < 0.05) in the BCON group compared to the CON group (Fig. 2A – C). However, ATLE supplementation mitigated (*P* < 0.05) the tBHP-induced reductions in the number of hierarchical follicles and the ovarian stroma index. Ovarian morphology based on H&E staining is presented in Fig. 2D. Compared with the CON group, ovaries from the BCON group contained more atretic follicles and exhibited necrotic inflammatory foci. These pathological changes were ameliorated in the BATLE group.

### Serum reproductive hormones

**Fig. 3:**
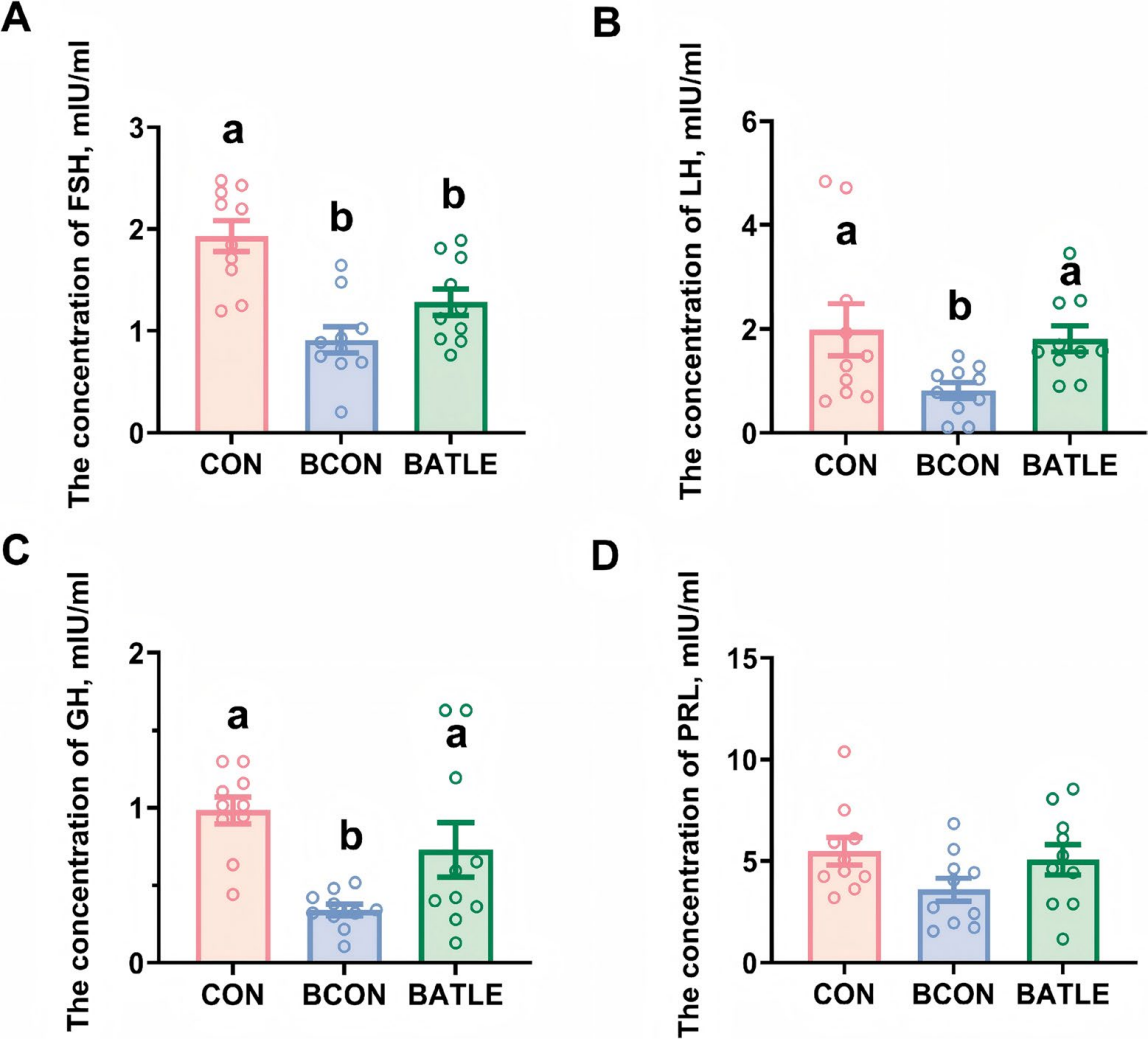
Effects of ATLE on serum hormone concentrations in tBHP-challenged hens. **A** FSH concentration, (**B**) LH concentration, (**C**) GH concentration, (**D**) PRL concentration. Data are expressed as mean ± standard error (SEM). Different superscripts in indicate a significant difference (*P* < 0.05). Abbreviations: ATLE, Acer truncatum Bunge leaf extract; FSH, follicle-stimulating hormone; LH, luteinizing hormone; GH, growth hormone; PRL, prolactin

As shown in Fig. 3, serum concentrations of FSH, LH, and GH were lower (*P* < 0.05) in the BCON group than in the CON group (Fig. 3A – C). In contrast, LH and GH concentrations in the BATLE group were higher (*P* < 0.05) than those in the BCON group. No differences (*P* > 0.05) in PRL concentrations were observed among the treatment groups (Fig. 3D).

### Antioxidant capacity

**Fig. 4:**
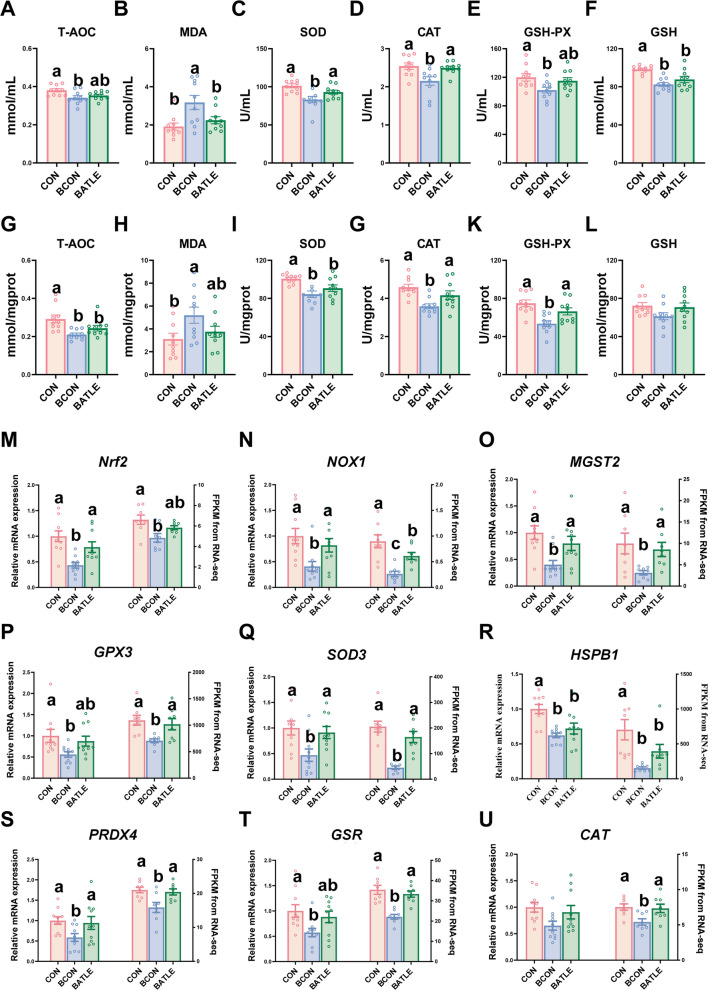
Effects of ATLE on antioxidant capacity and related gene expression in tBHP-challenged hens. **A** to **F** Antioxidant status of serum, including (**A**)T-AOC, (**B**) MDA, (**C**) SOD, (**D**) CAT, (**E**) GSH-Px, (**F**) GSH. **G** to **L** Antioxidant status of ovary, including (**G**) T-AOC, (**H**) MDA, (**I)** SOD, (**J**) CAT, (**K**) GSH-Px, and (**L**) GSH. **M** to **U** Antioxidant gene expression of ovary, including (**M**)*Nrf2*, (**N**)*NOX1*, (**O**) *MGST2*, (**P**) *GPX3*, (**Q**) *SOD3*, (**R**) *HSPB1*, (**S**) *PRDX4*, (**T**) *GSR*, (**U**) *CAT*. Data are expressed as mean ± standard error (SEM). Different superscripts indicate differences at *P* < 0.05. Abbreviations: ATLE, *Acer truncatum* Bunge leaf extract; T-AOC, total antioxidant capacity (expressed as Trolox equivalents); MDA, malondialdehyde; GSH, glutathione; GSH-Px, glutathione peroxidase; SOD, superoxide dismutase; CAT, catalase; *Nrf2*, nuclear factor E2-related factor 2; *NOX1*, NADPH oxidase 1; *HSPB1*, heat shock protein family B member 1; *MGST2*, microsomal glutathione S-transferase 2; *SOD3*, superoxide dismutase 3; *GPX3*, glutathione peroxidase 3; *GSR*, glutathione-disulfide reductase; *PRDX4*, peroxiredoxin 4; *CAT*, catalase

Antioxidant markers in serum and ovarian tissue are shown in Fig. 4A–L. In serum (Fig. 4A - F), the BCON group showed lower (*P* < 0.05) T-AOC, SOD activity, CAT activity, GSH-Px activity, and GSH concentration, but higher (*P* < 0.05) MDA concentration than the CON group. In contrast, the BATLE group exhibited higher (*P* < 0.05) SOD and CAT activities, and a lower (*P* < 0.05) MDA concentration than the BCON group. In ovarian tissue (Fig. 4G - L), the BCON group showed reduced (*P* < 0.05) T-AOC, SOD activity, CAT activity, and GSH-Px activity, along with an increased (*P* < 0.05) MDA concentration, relative to the CON group. However, ATLE supplementation mitigated (*P* < 0.05) the tBHP-induced decrease in CAT and GSH-Px activities and the increase in MDA concentration in the ovaries. For antioxidant-related genes, the ovarian mRNA expression levels of *NRF2*, *NOX1*, *MGST2*, *GPX3*, *SOD3*, *HSPB1*, *PRDX4*, and *GSR* were downregulated (*P* < 0.05) in the BCON group compared to the CON group (Fig. 4M – U). Importantly, under tBHP challenge, ATLE supplementation attenuated (*P* < 0.05) the downregulation of these antioxidant genes in ovaries. Changes in the expression of these genes were corroborated by RNA-seq FPKM values (Fig. 4M – U).

### Angiogenesis capacity

**Fig. 5:**
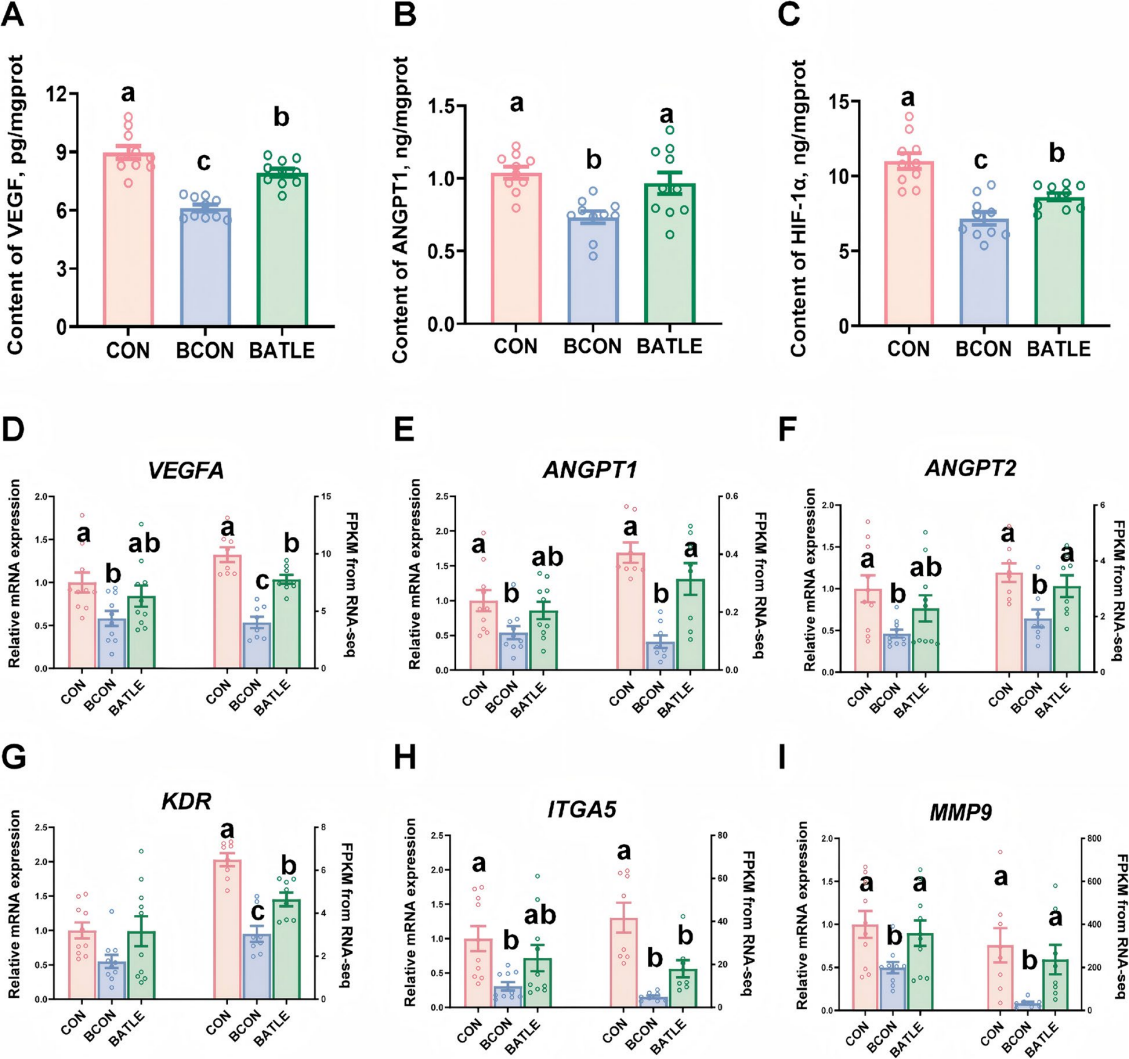
Effects of ATLE on angiogenic capacity and related gene expression in tBHP-challenged hens. (**A** to **C**) Angiogenesis status of ovary, including (**A**) VEGF, (**B**) ANGPT1, (**C**) HIF-1α. **D** to **I** Angiogenesis gene expression of ovary, including (**D**) *VEGFA*, (**E**) *ANGPT1*, (**F**) *ANGPT2*, (**G**) *KDR*, (**H**) *ITGA5*, (**I**) *MMP9*. Data are expressed as mean ± standard error (SEM). Different superscripts indicate differences at *P* < 0.05. Abbreviations: ATLE, *Acer truncatum* Bunge leaf extract; VEGF, vascular endothelial growth factor; ANGPT1, angiopoietin 1; HIF-1α, hypoxia inducible factor 1 subunit alpha; *VEGFA*, vascular endothelial growth factor A; *KD*R, vascular endothelial growth factor receptor 2; *ANGPT1*, angiopoietin 1; *ANGPT2*, angiopoietin 2; *MMP9*, matrix metallopeptidase 9; *ITGA5*, integrin alpha 5

The effect of ATLE on the angiogenic capacity of laying hens under oxidative stress is shown in Fig. 5. At the protein level, the ovarian contents of VEGF, ANGPT1, and HIF-1α were lower (*P* < 0.05) in the BCON group than in the CON group (Fig. 5A – C). Conversely, these protein levels were higher (*P* < 0.05) in the BATLE group than in the BCON group. At the gene expression level, the ovarian mRNA expression levels of *VEGFA*, *ANGPT1*, *ANGPT2*, *ITGA5*, and *MMP9* were downregulated (*P* < 0.05) in the BCON group relative to the CON group (Fig. 5D – I). ATLE supplementation attenuated (*P* < 0.05) the tBHP-induced downregulation of these angiogenesis-related genes. Consistent with these findings, changes in the expression of these angiogenesis-related genes (including *KDR*) were further supported by RNA-seq FPKM values (Fig. 5D – I).

### Ovarian transcriptome data overview

**Fig. 6:**
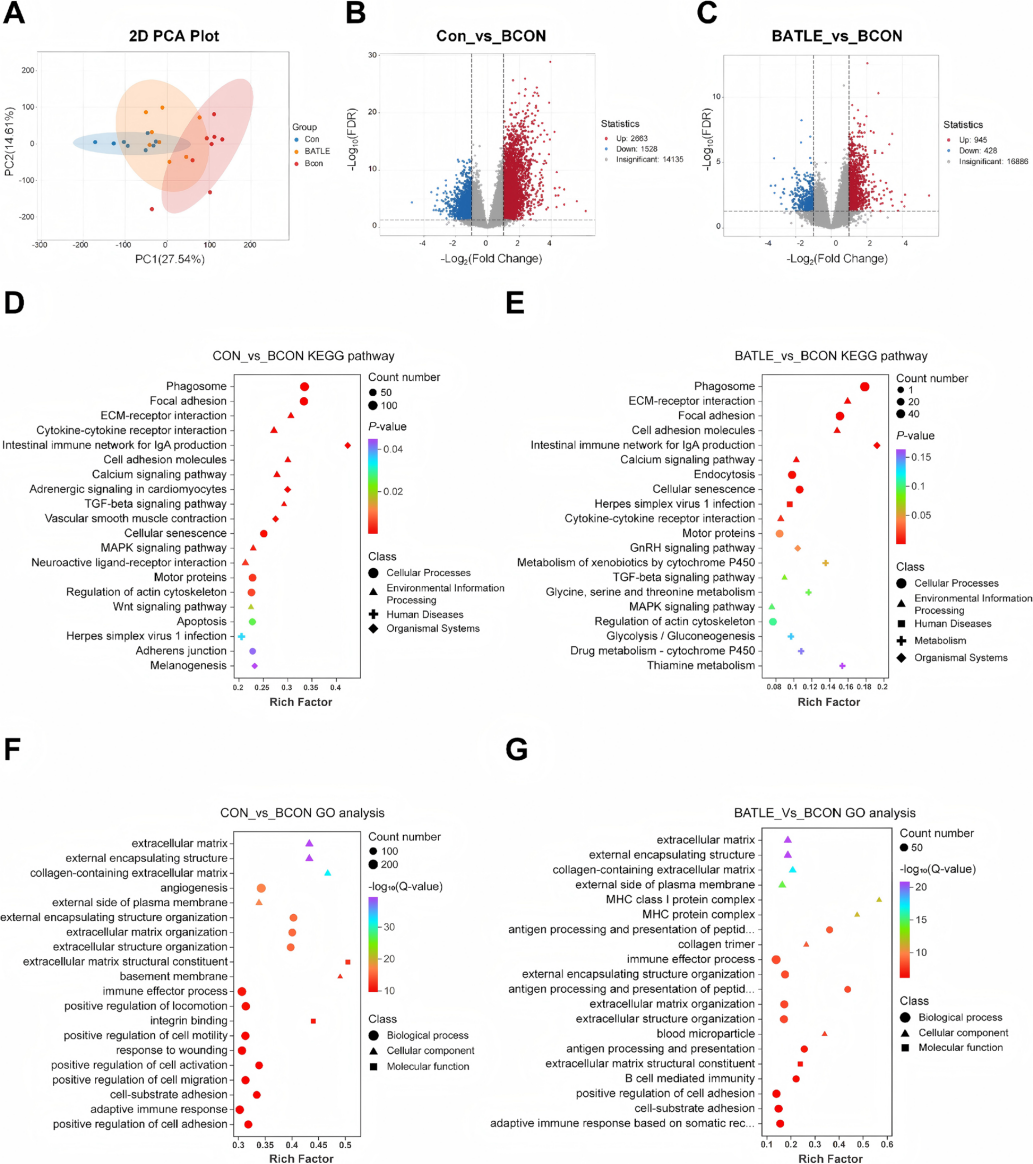
Overview of ovarian transcriptome statistics and functional enrichment analysis. **A** PCA score plot of groups, **B** Volcano maps in CON _vs_ BCON, **C** Volcano maps in BATLE _vs_ BCON, **D** to **E** Top 20 enrichment KEGG terms in CON _vs_ BCON and BATLE _vs_ BCON, **F** to **G** Top 20 enrichment GO terms in CON _vs_ BCON and BATLE _vs_ BCON. Abbreviations: ATLE, *Acer truncatum* Bunge leaf extract; PCA, principal component analysis; KEGG, Kyoto Encyclopedia of Genes and Genomes; GO, gene ontology

Transcriptomic profiling results are presented in Fig. 6. PCA revealed a clear separation between the CON and BCON groups along PC1 (27.54% variance) and PC2 (14.61% variance). The BATLE group clustered intermediately, with its confidence ellipse partially overlapping both groups (Fig. 6A). Differential expression analysis identified 4,191 genes (2,663 up- and 1,528 down-regulated) in the BCON vs. CON comparison (Fig. 6B) and 1,373 genes (945 up- and 428 down-regulated) in the BATLE vs. BCON comparison (Fig. 6C). The detailed lists of differentially expressed genes (DEGs) for both comparisons are provided in Supplementary Table 1. KEGG pathway analysis revealed that in the CON vs. BCON comparison, the DEGs were enriched in pathways related to immune response and structural integrity, including Phagosome, Focal adhesion, ECM-receptor interaction, and Cytokine-cytokine receptor interaction (Fig. 6D). Interestingly, similar pathways, including Phagosome, ECM-receptor interaction, and Focal adhesion, were also enriched in the BATLE vs. BCON comparison (Fig. 6E). GO enrichment analysis provided further insights. In the CON vs. BCON comparison, biological processes (BP) related to immune effector process, response to wounding, and angiogenesis were enriched, while cellular components (CC) were concentrated in the extracellular matrix and collagen-containing extracellular matrix (Fig. 6F). In the BATLE vs. BCON comparison, CC functional categories including extracellular matrix, collagen trimer, and basement membrane were enriched. Furthermore, BP functional categories associated with adaptive immunity, such as antigen processing and presentation, MHC protein complex, and B cell-mediated immunity, were also enriched (Fig. 6G).

### Ovarian transcriptome integration analysis

**Fig. 7:**
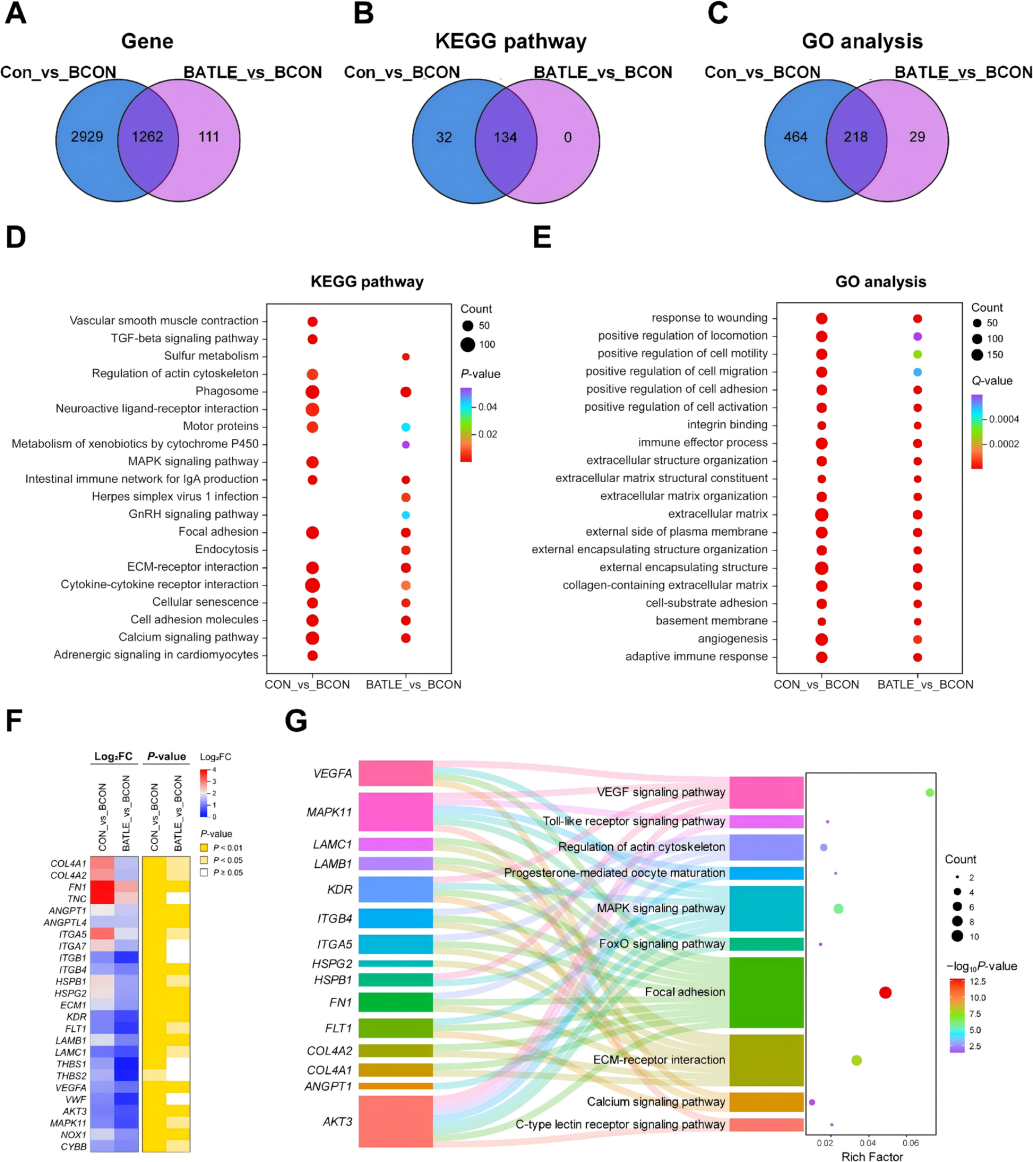
Integrative analysis of differentially expressed genes and related pathways. **A** Venn analysis of DEGs, **B** Venn analysis of KEGG terms, **C** Venn analysis of GO terms, **D** Co-enrichment KEGG plot, **E** Co-enrichment GO plot, **F** Heatmap of DEGs in angiogenesis, **G** Sankey bubble chart of DEGs in angiogenesis. Abbreviations: ATLE, Acer truncatum Bunge leaf extract; DEGs, differentially expressed genes; KEGG, Kyoto Encyclopedia of Genes and Genomes; GO, Gene Ontology

Venn diagram analysis of DEGs, KEGG pathways, and GO terms indicated a substantial overlap between the two comparisons (Fig. 7A-C). At the gene level, 1,262 DEGs were shared, accounting for 91.9% of all DEGs in the BATLE vs. BCON comparison (Fig. 7A). All 134 KEGG pathways enriched in the BATLE vs. BCON comparison were also enriched in the CON vs. BCON comparison (Fig. 7B). Similarly, 218 GO terms were shared, representing 88.2% of the terms modulated by ATLE (Fig. 7C). Co-enrichment analysis of these shared functional terms highlighted consistent patterns (Fig. 7D, E). Key overlapping KEGG pathways included Phagosome, Focal adhesion, and ECM-receptor interaction. Similarly, overlapping GO terms were consistently related to tissue structure and vascular dynamics, such as extracellular matrix, angiogenesis, and response to wounding. To validate the central role of angiogenesis and extracellular matrix remodeling suggested by the enrichment analyses, the expression levels of key related genes were further examined (Fig. 7F). Genes crucial for angiogenesis (e.g., *VEGFA*, *FLT1*, and *KDR*), *ECM compositio*n (e.g., *COL1A1*, *COL4A2*, *FN1*, *LAMC1*), focal adhesion (e.g., *ITGA5*, *ITGA7*), and those linking oxidative stress and angiogenesis (e.g., *HSPB1*) followed the same trend of down-regulation by tBHP and up-regulation by ATLE. Key molecules, including *VEGFA*, *KDR*, *ITGA5*, *HSPB1*, and *FN1*, converged upon pivotal pathways, including the VEGF signaling pathway, Focal adhesion, and ECM-receptor interaction (Fig. 7G).

## Discussion

Consistent with prior work showing that oxidative stress impaired poultry performance, tBHP exposure reduced (*P* < 0.05) both egg-laying rate and feed conversion efficiency in laying hens (Ding et al. 2022; Wang et al. 2022). Supplementing the diet with 0.6% ATLE largely prevented this decline, indicating that ATLE could stabilize production under oxidative challenge. This improvement in performance coincided with preservation of ovarian morphology (Tao et al. 2022; Ma et al. 2024). Histological analysis showed that ATLE attenuated (*P* < 0.05) the loss of hierarchical follicles and prevented the decline in the ovarian stroma index, thus maintaining the structural substrate required for continued ovulation. Because follicular development depends on endocrine regulation and oxidative stress disrupts hypothalamic–pituitary–gonadal homeostasis, related hormones were measured (Cheng et al. 2021; Ru et al. 2024; Wang et al. 2021a, b). tBHP exposure lowered (*P* < 0.05) serum FSH, LH, and GH, which would have impeded follicle recruitment and dominance; ATLE treatment restored these hormone concentrations. Altogether, these results indicated that ATLE protected ovarian structure and reestablished endocrine balance, permitting normal follicle development and maturation and thereby preventing declines in production performance.

Oxidative stress was widely considered a major driver of ovarian functional decline in laying hens (Ru et al. 2024). Consistent with previous reports, tBHP exposure disrupted redox homeostasis in laying hens, as evidenced by increased MDA concentration and reduced activities of antioxidant enzymes (SOD, CAT, GSH-Px) (Wang et al. 2021a, b; Ding et al. 2022). These alterations promoted granulosa cell apoptosis and increased follicular atresia. Building on a prior observation that ATLE had antioxidant activity in a broiler model, the present study showed that ATLE reversed ovarian oxidative damage markers in laying hens (Liu et al. 2023). Molecular analyses indicated that ATLE’s protective effect was associated with activation of the Nrf2 signaling pathway. Nrf2 is a central regulator of the cellular antioxidant stress response (Karunatilleke et al. 2021). Once activated, it could translocate to the nucleus and initiate the transcription of downstream cytoprotective genes (Hirotsu et al. 2012; Deshmukh et al. 2017). Both qPCR and RNA-seq consistently demonstrated that ATLE upregulated (*P* < 0.05) *NRF2* and downstream targets (e.g., *SOD3*, *GPX3*, *HSPB1*, *PRDX4*), thereby enhancing ROS scavenging capacity. These results were consistent with reports that plant extracts, including theabrownin and resveratrol, preserved reproductive function through Nrf2 (Wang et al. 2024; Zhang et al. 2024; Brouklogiannis and Mountzouris 2025). It was therefore proposed that ATLE’s active constituents (likely flavonoids and polyphenols) exerted antioxidant effects either by directly scavenging ROS or by modulating the KEAP1–Nrf2 interaction.

A second novel finding of this study was that ATLE preserved ovarian angiogenesis in addition to providing antioxidant protection. Follicle recruitment and growth require vascular expansion to deliver nutrients and hormones (Li et al. 2022; Travisano and Lien 2025), and insufficient angiogenesis is a major contributor to follicular atresia (Li et al. 2020; Wang et al. 2025). tBHP exposure suppressed (*P* < 0.05) key angiogenic factors, including VEGFA, ANGPT1, and KDR, which indicated that oxidative stress could impair endothelial function or disrupt hypoxia signaling (such as HIF-1α transduction) and thereby cause microcirculatory disorders and loss of hierarchical follicles (Zhao et al. 2024; Jiang et al. 2025a, b). ATLE supplementation reversed the suppressive effects of tBHP; in particular, the increases (*P* < 0.05) in VEGF, ANGPT1, and HIF-1α protein levels, together with the upregulation (*P* < 0.05) of angiogenesis-related transcripts such as VEGFA and ANGPT1, suggested that ATLE promoted reconstruction of a functional vascular network in the ovary. These findings were consistent with the known biological actions of the active components present in similar plant extracts. Previous studies reported that resveratrol stimulated endothelial cell migration and tubular structure formation via the SIRT1/HIF-1α pathway (Tino et al. 2016; Nishigaki et al. 2022; Wang et al. 2024). Quercetin promoted angiogenesis and protected the blood-spinal cord barrier structure after spinal cord injury by targeting the PI3K/Akt signaling pathway (Liu et al. 2025a, b, c). Lycopene protected corneal endothelial cells from oxidative stress by regulating the p62-autophagy-keap1/nrf2 pathway (Liu et al. 2025a, b, c). Ginsenosides promoted endothelial proliferation and increased physiological microvascular density through the PI3K/Akt and eNOS pathways (Chen et al. 2019). Tanshinone IIA, epicatechin, and icariin protected endothelial cells from oxidative damage and thereby preserved vascular regenerative capacity (Feng et al. 2021; Yan et al. 2021; Li et al. 2024a, b). Because ATLE is rich in similar active ingredients, it was proposed that its effects extended beyond passive antioxidant defense to active regulation of ovarian microcirculatory homeostasis. Mechanistically, ATLE appeared to protect vascular endothelial cells from ROS-mediated dysfunction and thus provided a stable structural basis for follicular development.

Transcriptomic analysis showed that ECM pathways and immune processes were consistently the main targets of tBHP-induced injury and of ATLE-mediated rescue. Crucially, these transcriptomic findings supported and expanded upon the targeted gene expression data. While RT-qPCR confirmed the targeted activation of the Nrf2 antioxidant defense and VEGF/ANGPT1-mediated angiogenesis, the RNA-seq data revealed that these specific protective mechanisms were functionally coupled with broader microenvironmental remodeling. For instance, the transcriptomic enrichment of ECM-receptor interaction and focal adhesion pathways provided the macroscopic structural context required for the targeted angiogenic factors to support endothelial cell migration and vascular network reconstruction. Specifically, tBHP-induced oxidative stress caused downregulation of genes encoding key ECM components, such as collagens (*COL1A1*, *COL4A2*), laminin (*LAMC1*), fibronectin (*FN1*), and the matrix metalloproteinase *MMP9* (Hu et al. 2025; Qiu et al. 2024). At the same time, angiogenic factors (*VEGFA*, *ANGPT1*) and the endothelial‑stability regulator *HSPB*1 were suppressed (Chen et al. 2024a, b; Li et al. 2025a, b). These changes indicated disruption of the ovarian vascular microenvironment and of the tissue’s physical scaffold, which would impair the biochemical signaling required for cell survival and differentiation (Xu et al. 2022a, b; Shi et al. 2023). ATLE treatment upregulated the above genes and *ITGA5* (Xu et al. 2023), reversing the molecular damage. This molecular recovery matched the pathway enrichment for wound response and focal adhesion (Li et al. 2024a, b; Jain et al. 2025), and supported the conclusion that ATLE restored the adhesive interactions necessary for endothelial cell migration. By rebuilding the ECM scaffold and promoting physiological angiogenesis, ATLE provided a structural basis for follicle growth and restoration of egg‑laying performance.

In summary, the present study demonstrated that ATLE mitigated tBHP-induced oxidative stress in laying hens through the coordinated regulation of antioxidant defenses, angiogenesis, and microenvironment remodeling. Dietary ATLE reversed the decline in egg-laying rate and alleviated reproductive endocrine disturbances caused by oxidative damage, while improving follicular development and ovarian histology. The protective effect of ATLE was multi-targeted: it enhanced systemic antioxidant capacity by activating the Nrf2 signaling pathway and promoted ovarian angiogenesis via the VEGF/ANGPT1 signaling axis, thereby preserving tissue homeostasis. Moreover, transcriptomic analysis offered an additional mechanistic insight, indicating that ATLE regulated extracellular matrix (ECM) remodeling and modulated the immune microenvironment. These results provided a theoretical rationale for using ATLE as a multi-targeted, plant-derived additive to improve reproductive health in laying hens.

This study showed that ATLE had both antioxidant and pro-angiogenic effects, but several limitations remained. First, due to the acute nature of the tBHP-induced chemical stress model, the trial was conducted over a relatively short period (4 weeks) with a focused sample size (n = 10 per group). While this design provided sufficient statistical power to elucidate molecular and physiological mechanisms under acute stress, it had limitations in evaluating long-term, large-scale commercial production performance. Therefore, the relevance of these results to commercial production environments requires further validation. Second, ATLE is a complex mixture, and the specific contributions of individual active compounds to the observed effects were not identified. Finally, the mechanism by which ATLE promoted angiogenesis remained unresolved. The possibility that increased angiogenesis was a secondary outcome of reduced oxidative stress rather than a direct action of ATLE could not yet be excluded (Kweider et al. 2011; Chen et al. 2024a, b). Future studies should use vascular endothelial cell models to determine whether ATLE stimulates angiogenesis directly or acts indirectly by mitigating oxidative damage (Dubey et al. 2025; Jiang et al. 2026; Zhou et al. 2025).

## Conclusion

Dietary supplementation with ATLE reduced tBHP-induced oxidative stress in laying hens and restored production performance. This benefit was achieved through activation of the Nrf2 antioxidant pathway, promotion of VEGF-mediated angiogenesis, and ECM remodeling, which collectively repaired ovarian morphology and improved reproductive hormone homeostasis. These findings provided a theoretical foundation for using ATLE as a natural feed additive that combines antioxidant action with microenvironment regulation to improve poultry reproductive health, highlighting the importance of preserving the ovarian vascular microenvironment in anti-stress strategies for laying hens.

## Materials and methods

### Animal ethics statement

The protocol for the experimental animal procedures was approved by the Institutional Animal Care and Use Committee of Northwest A&F University (Permit Number: NWAFAC 1008). Researchers sought to provide a suitable habitat for the laboratory animals.

### Experiment design and management

ATLE was obtained from the Shaanxi Huike Plant Development Co. Ltd. (Xi'an, China). To investigate the effects of ATLE supplementation on laying hens under oxidative stress, a total of 30 healthy Hy-Line Brown hens (initial body weight = 1894.3 ± 94.2 g, laying rate = 86.7% ± 5.5%) were randomly divided into three experimental groups: (1) CON group (basal diet and saline injection); (2) BCON group (basal diet and 800 μmol/kg BW tBHP injection); and (3) BATLE group (0.6% ATLE-supplemented diet +800 μmol/kg BW tBHP injection). Each group consisted of 10 replicates, with one hen per replicate (n = 10). All hens were housed individually in separate wire cages (40 ×45 ×45 cm) equipped with independent feeders and nipple drinkers. The trial lasted for 28 days. The doses of tBHP and ATLE were selected according to previous studies (Ding et al. 2022; Liu et al. 2023). Specifically, hens in the BCON and BATLE groups were injected intraperitoneally with 800 μmol/kg BW of tBHP every 4 days throughout the 28-day trial (Ding et al. 2022; Liu et al. 2023; Wang et al. 2021a, b). The tBHP was diluted in sterile physiological saline to a constant injection volume of 3 mL per hen. To ensure accuracy, the injection dose was calculated and dynamically adjusted based on the body weight of each hen recorded weekly. The CON group received an equivalent volume (3 mL) of sterile saline intraperitoneally following the same 4-day schedule.

For production performance analysis, egg production, egg weight, and feed consumption were recorded daily throughout the 28-day experimental period. The laying rate, average egg weight, average daily feed intake (ADFI), and feed conversion ratio (FCR) were calculated based on the cumulative data from day 1 to day 28. The FCR was calculated as follows: FCR = total feed intake/total egg weight. The experimental design is illustrated in the graphical abstract. The basal diet was formulated to meet the nutritional requirements recommended by the Hy-Line Brown Commercial Management Guide (2022) and the formulation and nutrient composition of the basal diet are shown in Table S1.

### Ovarian sample collection and morphology

At the conclusion of the trial (day 28, 48 h after the final tBHP injection), following an overnight fast of 12 h (with free access to water), blood samples were collected from the wing vein of all hens (*n* = 10 per group). Serum was immediately separated by centrifugation (3,000 × g, 10 min) in serum collection tubes and stored for subsequent analyses. The hens were then euthanized by exsanguination, and the ovaries were immediately separated and collected. Ovarian tissues were promptly processed to isolate and enumerate hierarchical and preovulatory (≥ 4 mm) follicles. The remaining ovarian stroma was weighed to calculate the organ index. For histological evaluation, ovarian tissues were fixed in 4% paraformaldehyde for 24 h, followed by paraffin embedding and sectioning at 4 μm thickness. Tissue sections were stained with hematoxylin and eosin (H&E) according to standard protocols and visualized using a light microscope.

### Serum reproductive hormones

Serum concentrations of follicle-stimulating hormone (FSH, Cat. No. WLCSJZF60058), luteinizing hormone (LH, Cat. No. WLCSJZF60197), growth hormone (GH, Cat. No. ED-60127), and prolactin (PRL, Cat. No. WLCSJZF69271) were measured using commercial assay kits (LunChangShuo, Shanghai, China), according to the manufacturer's instructions.

### Antioxidant and angiogenesis markers

For ovarian tissue samples, a 10% ovarian tissue homogenate was prepared by adding homogenate buffer with a weight to volume ratio of 1:9. The mixture was centrifuged for 10 min (4,000 × g) at 4 °C, and the supernatant was collected for later use. Antioxidant capacity analysis was performed using commercial kits (Nanjing Jiancheng Bioengineering Institute, Nanjing, China) according to the manufacturer's instructions, including malondialdehyde (MDA, Cat. No. A003-1–2), total antioxidant capacity (T-AOC, Cat. No. A015-2–1), superoxide dismutase (SOD, Cat. No. A001-3–2), catalase (CAT, Cat. No. A007-1–1), glutathione (GSH, Cat. No. A006-1–1), and glutathione peroxidase (GSH-Px, Cat. No. A005-1–2). Angiogenesis markers were determined using ELISA kits (LunChangShuo, Shanghai, China), including vascular endothelial growth factor (VEGF, Cat. No. ED-60148), angiopoietin 1 (ANGPT1, Cat. No. ED-63176), and hypoxia inducible factor 1 subunit alpha (HIF-1α, Cat. No. ED-60538).

### Ovarian gene expression by real-time PCR

Nine antioxidant-related genes (*Nrf2*, *NOX1*, *HSPB1*, *SOD3*, *GPX3*, *MGST2*, *PRDX4*, *GSR*, and *CAT*) and six angiogenesis-related genes (*VEGFA*, *ANGPT1*, *ANGPT2*, *KDR*, *ITGA5*, *MMP9*) were analyzed using RT-qPCR. Gene-specific primers were designed based on Gallus gallus gene sequences. Primer sequences were listed in Table S2. For RNA preservation, the collected ovarian tissues were immediately snap-frozen in liquid nitrogen and stored at −80 °C. Total RNA was extracted from the ovarian tissue using the TRIZOL kit protocol (AG21101, Agbio, China), and the quality and concentration of the RNA were assessed using the NanoDrop 2000 (Thermo Fisher Scientific, Waltham, MA). The cDNA was synthesized using the Primer Script RT Reagent Kit (TaKaRa, Dalian, China), following the manufacturer's recommended protocol. Then RT-qPCR was performed using SYBR green mix (Accurate Biology, Changsha, China) on the cDNA samples. *β-actin* was used as an internal reference gene, and the relative expression of each gene was calculated using the 2^−ΔΔCT^ method.

### Ovarian RNA-Seq experiment and data analysis

The total RNA extracted as described above was identified and quantified using a Qubit fluorescence quantifier (Life Technologies, CA, USA) and a Qsep400 high-throughput biofragment analyzer (Bioptic Inc., Taiwan, China). The qualified RNA samples were used to generate a library. After the initial library was constructed, the Qubit fluorescence quantifier and Qsep400 high-throughput biofragment analyzer were used to evaluate the library quality. The cDNA libraries were sequenced on the Illumina sequencing platform by Metware Biotechnology Co., Ltd. (Wuhan, China).

The raw data were filtered with FastQC (v0.23.2), then the clean reads were aligned against the reference genome using HISAT2 (v2.2.1). Gene read counts were determined using featureCounts (v2.0.3), and expression levels were normalized using the Fragments Per Kilobase of transcript per Million mapped reads (FPKM) method. DESeq (v1.22.1) was utilized for differential expression analysis, calculating fold change (FC) values between groups. Differentially expressed genes were identified based on the criteria |log₂FC|> 1 and adjusted *P*-value < 0.05. Principal Component Analysis (PCA) score plot, volcano map, Venn analysis, heatmap, Kyoto Encyclopedia of Genes and Genomes (KEGG) analysis, and Gene Ontology (GO) enrichment analysis were performed using the Metware Cloud (https://cloud.metware.cn).

### Statistical analysis

Data were analyzed by one-way ANOVA, followed by Duncan's multiple comparison test, using IBM SPSS Statistics 22. The individual hen was considered the experimental unit for all analyses. Specifically, the sample size was n = 10 per treatment group for production performance, organ indices, serum parameters, and RT-qPCR analyses. The results were presented as arithmetic mean and SEM, and a threshold of *P* < 0.05 was used for statistical comparisons. Figures were created using GraphPad Prism 8.0 or Metware Cloud (https://cloud.metware.cn).

## Supplementary Information

Supplementary Material 1.

Supplementary Material 2.

## Data Availability

The composition and nutritional profiles of the experimental diets, along with the forward and reverse primer sequences used for PCR analysis, are provided in the Supplementary Materials. Further details regarding the transcriptomic analysis are also included therein.

## Abbreviations

ADFI: Average daily feed intake
ANGPT1: Angiopoietin 1
ANGPT2: Angiopoietin 2
ATLE: *Acer truncatum* Bunge leaf extract
CAT: Catalase
DEGs: Differentially expressed genes
FC: Fold change
FCR: A ratio of the feed intake to the egg weight
FPKM: Fragments per kilobase of transcript per million mapped reads
FSH: Follicle-stimulating hormone
GH: Growth hormone
GO: Gene Ontology
GPX3: Glutathione peroxidase 3
GSH: Glutathione
GSH-Px: Glutathione peroxidase
GSR: Glutathione-disulfide reductase
H&E: Hematoxylin and eosin staining
HIF-1α: Hypoxia inducible factor 1 subunit alpha
HSPB1: Heat shock protein family B member 1
ITGA5: Integrin alpha 5
KDR: Vascular endothelial growth factor receptor 2
KEGG: Kyoto encyclopedia of genes and genomes
LH: Luteinizing hormone
MDA: Malondialdehyde
MGST2: Microsomal glutathione S-transferase 2
MMP9: Matrix metallopeptidase 9
NOX1: NADPH oxidase 1
Nrf2: Nuclear factor E2-related factor 2
PCA: Principal Component Analysis
PRDX4: Peroxiredoxin 4
PRL: Prolactin
ROS: Reactive oxygen species
SOD: Superoxide dismutase
SOD3: Superoxide dismutase 3
T-AOC: Total antioxidant capacity
tBHP: Tert-butyl hydroperoxide
VEGF: Vascular endothelial growth factor
VEGFA: Vascular endothelial growth factor A
