# Supplementary material for: Dietary supplementation of Acer truncatum leaf extract alleviates oxidative-stress induced impairment of ovarian function in laying hens via Nrf2-mediated antioxidant defense and VEGF-mediated angiogenesis
Source: Stress Biol. 2026 Aug 12;6(1):60. doi: 10.1007/s44154-026-00329-x (PMC13462015; doi:10.1007/s44154-026-00329-x)
Supplement: Supplementary file 1 [file 44154_2026_329_MOESM1_ESM.docx]

**Table S1.** Ingredients and nutrient levels of the basal diet (as feed basis)

| Item | %, unless noted |
| --- | --- |
| Ingredients |  |
| Corn | 59.76 |
| Soybean meal | 23.00 |
| Distillers dried grains with solubles | 4.00 |
| Calcium carbonate | 8.65 |
| Dicalcium phosphate | 1.05 |
| Soybean oil | 1.25 |
| Sodium chloride | 0.25 |
| L-Lysine-H2SO4 | 0.13 |
| L-Threonine | 0.31 |
| DL-Methionine | 0.25 |
| Choline chloride | 0.10 |
| Sand | 0.25 |
| Premix^1^ | 1.00 |
| Total | 100.0 |
| Nutrient levels |  |
| Metabolizable energy (calculated), kcal/kg | 2600 |
| Crude protein (calculated/analyzed) | 16.5/16.4 |
| Total phosphorus (calculated/analyzed) | 0.51/0.48 |
| Calcium (calculated/analyzed) | 3.50/3.65 |
| Available phosphorus (calculated)^2^ | 0.36 |
| Lysine^2^ | 0.86 |
| Methionine (calculated)^2^ | 0.51 |
| Methionine + Cystine (calculated)^2^ | 0.80 |
| Threonine (calculated)^2^ | 0.91 |

^1^ The premix provided the following per kilogram of diets: vitamin A 8000 IU, vitamin D_3_ 1600 IU, vitamin E 30 mg, vitamin K_3_ 1.5 mg, vitamin B_1_ 4 mg, vitamin B_2_ 13 mg, vitamin B_6_ 6 mg, vitamin B_12_ 0.02 mg, biotin 0.15 mg, pantothenic acid 15 mg, nicotinic acid 20 mg, folic acid 1.5 mg, Cu (as copper sulfate) 8 mg, Fe (as ferrous sulfate) 80 mg, Mn (as manganese sulfate) 60 mg, Zn (as zinc sulfate) 80 mg, I (as potassium iodide) 0.35 mg, Se (as sodium selenite) 0.3 mg, choline chloride 800 mg, phytase 200 mg.

^2^ Nutritional values were calculated based on the Tables of Feed Composition and Nutritive Values in China (2020, 31st Edition).

**Table S2.** Primers used for RT-qPCR

| Genes^1^ | Prime sequence (5′-3′) | NCBI number |
| --- | --- | --- |
| *Nrf2* | F: AGAAAACGCTGAACCACCAATC | NM_205117.2 |
|  | R: GCTGGGTGGCTGAGTTTGATTA |  |
| *NOX1* | F: TGGACGGAGCACATCATTG | NM_001101830.3 |
|  | R: AGGCAAGCAGGTCATTGAAC |  |
| *HSPB1* | F: GAGATCACCGGCAAACACG | NM_205290.2 |
|  | R: TGATCTCGGATGACTGGATG |  |
| *MGST2* | F: TGACTTAGCTCTGCTCGCTG | XM_001234229.7 |
|  | R: CCAACGTTGAAGCCCAGGTA |  |
| *SOD3* | F: TTGTGATCCATGAGCAGGAA | XM_040669203.2 |
|  | R: TTGTTGCAGATCCCAATCAC |  |
| *GPX3* | F: AGGAGTACATCCCCTTCCGA | NM_001163232.3 |
|  | R: TAGGGCCCCAGCTCATTTG |  |
| *GSR* | F: TCCTGACTACGGCTTCGAGA | XM_015276627.4 |
|  | R: AACTTGCCGTAACCACGGAT |  |
| *PRDX4* | F: GCCAGGCCAAGATCTCGAA | XM_046912353.1 |
|  | R: AGCAGGGCAAACTTCTCCAT |  |
| *CAT* | F: GGTTCGGTGGGGTTGTCTTT | NM_001031215.2 |
|  | R: CACCAGTGGTCAAGGCATCT |  |
| *VEGFA* | F: GAAGCCCAACGAAGTTATCAAA | NM_205042.3 |
|  | R: ACATCCACAGGGACACATTCTA |  |
| *KDR* | F: CTGAAGGTGCACTCCTCCTC | NM_001004368.2 |
|  | R: CAAGTATGGCTCAACGCAGA |  |
| *ANGPT1* | F: ACAAAAGCGGCGTCTACACT | NM_001199447.4 |
|  | R: AGCCAGTGTTCACCTGATGG |  |
| *ANGPT2* | F: ATGCTTGTGGTCCTTCTAACC | NM_204817.2 |
|  | R: TCCTGAGCCTTTCCAGTAGTA |  |
| *MMP9* | F: ACCTGGACCGTGCCGTGATAG | NM_204667.2 |
|  | R: CTGCCTCGCCGCTGTAAATCTG |  |
| *ITGA5* | F: CTCCAACTACCCCGAGTACT | XM_046904986.1 |
|  | R: CACCGAATAGCCCATATAAC |  |
| *β-actin* | F: AATCAAGATCATTGCCCCACCT | NM_205518.1 |
|  | R: TGGGTGTTGGTAACAGTCCG |  |

^1^ Abbreviation represents: *Nrf2*, nuclear factor E2-related factor 2; *NOX1*, NADPH oxidase 1; *HSPB1*, heat shock protein family B member 1; *MGST2*, microsomal glutathione S-transferase 2; *SOD3*, superoxide dismutase 3; *GPX3*, glutathione peroxidase 3; *GSR*, glutathione-disulfide reductase; *PRDX4*, peroxiredoxin 4; *CAT*, catalase; *VEGFA*, vascular endothelial growth factor A; *KD*R, vascular endothelial growth factor receptor 2; *ANGPT1*, angiopoietin 1; *ANGPT2*, angiopoietin 2; *MMP9*, matrix metallopeptidase 9; *ITGA5*, integrin alpha 5.
